# Remarkable Response to Methylprednisolone in a Multiple Myeloma Patient with Nodal Disease Refractory to High-Dose Chemotherapy

**DOI:** 10.7759/cureus.411

**Published:** 2015-12-16

**Authors:** Kimberly Celotto, Vishala Neppalli, Sarah Holstein

**Affiliations:** 1 Medicine, Roswell Park Cancer Institute; 2 Pathology, Roswell Park Cancer Institute

**Keywords:** Multiple Myeloma, corticosteroid, extramedullary, autologous stem cell transplantation, nodal metastases

## Abstract

Multiple myeloma is a disorder of malignant plasma cells, which is characterized by paraprotein production, lytic bone lesions, hypercalcemia, susceptibility to infections, and renal impairment. Here, we describe a patient with IgA myeloma with extensive nodal involvement who obtained significant benefit from high-dose methylprednisolone after failing aggressive induction chemotherapy and high-dose melphalan with autologous stem cell support.

## Introduction

Multiple myeloma is a hematological malignancy involving clonal plasma cells, which is characterized by monoclonal protein production, lytic bone disease, immune suppression, anemia, and renal impairment. Multiple myeloma is primarily a bone marrow malignancy; however, extramedullary disease can arise in the form of plasmacytomas. For patients who are eligible, the backbone of therapy remains induction therapy with multiple agents followed by consolidation with high-dose melphalan and autologous stem cell transplant. Despite the marked improvements in outcomes with newer agents used in combination with the transplant, the majority of patients will inevitably relapse. Whether patients with extensive extramedullary involvement require different treatment strategies has yet to be determined.

## Case presentation

**Figure 1:**
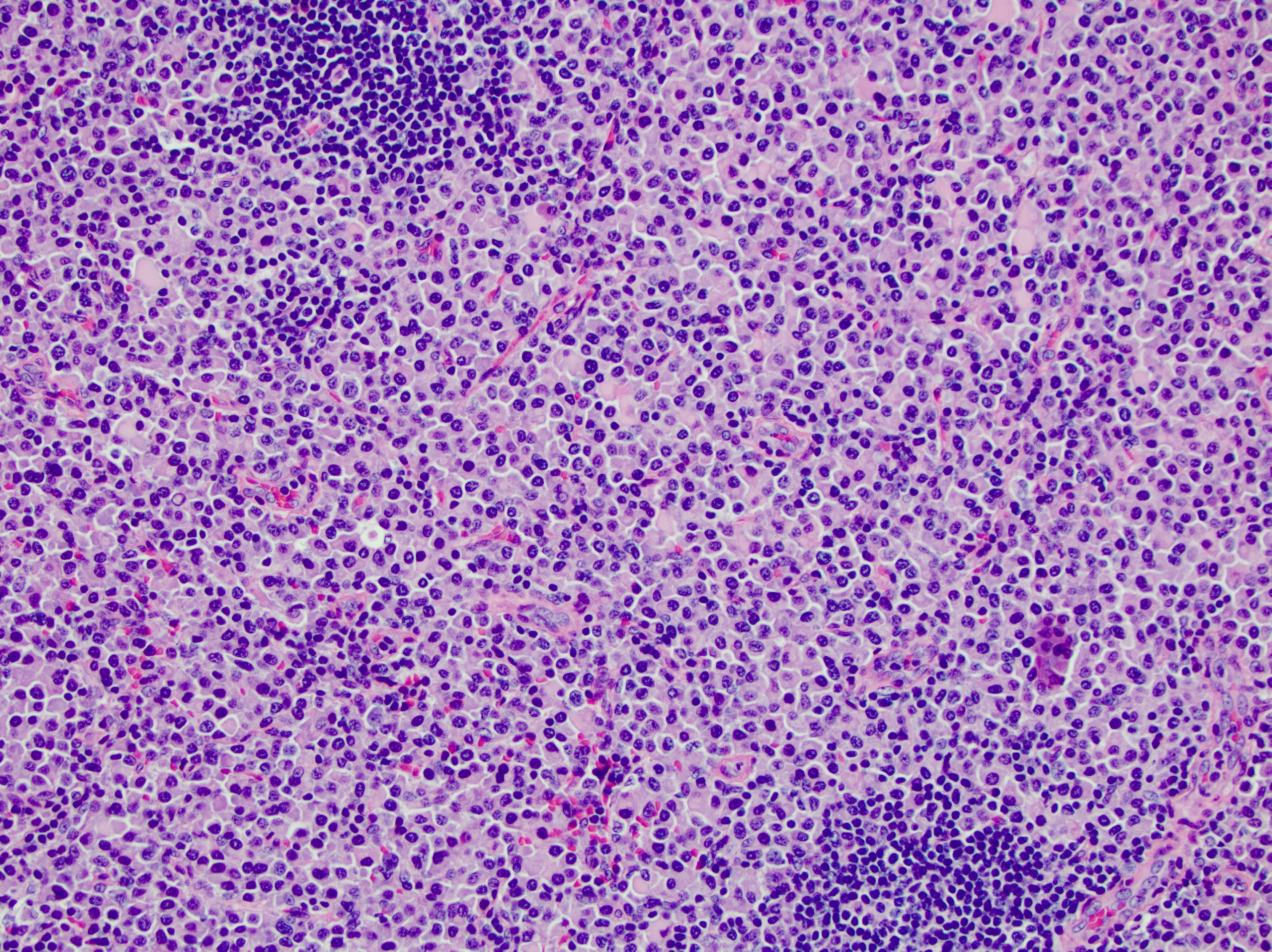
Inguinal lymph node biopsy demonstrates sheets of plasma cells which efface the nodal architecture (200X magnification). Residual germinal cell clusters are present in the left upper and right lower corners of the image.

A 57-year-old Caucasian female with a past medical history notable only for chronic back pain presented in early 2013 for an MRI of her spine. No abnormalities were observed in the spine; however, diffuse lymphadenopathy was noted in the retroperitoneum. She subsequently underwent a CT of the abdomen/pelvis, which showed extensive adenopathy. PET/CT showed moderate uptake in adenopathy involving the neck, thorax, abdomen, and pelvis. Informed patient consent was obtained for her treatment. A biopsy of a left groin node was performed which showed diffuse effacement of the lymph node architecture by sheets of medium-size plasma cells (Figure 1). The neoplastic cells were positive for CD45, CD79a, MUM-1, Bcl-2, kappa light chain, and IgA, and negative for CD20, CD22, CD3, CD138, Pax-5, CD10, Bcl-6, CD5, CD56, Bcl-1, IgG, EBV-LMP, and ALK-1. The MIB-1 proliferation index was approximately 5% in the neoplastic cells. Overall, these results were consistent with a plasma cell neoplasm involving the lymph node.

She subsequently underwent workup for multiple myeloma in June 2013. Laboratory studies were notable for serum creatinine 0.82 mg/dL, albumin 4.1 g/dL, hemoglobin 13.6 g/dL, b2-microglobulin of 7.65, IgG 1530 mg/dL, IgA 2740 mg/dL, IgM 33 mg/dL, serum kappa 160 mg/L, and serum lambda 29.6 mg/L. SIFE showed a monoclonal IgA kappa protein and SPEP showed an M-spike of 1.37 g/dL in the beta region and 0.2 g/dL in the gamma region. A bone marrow biopsy showed trilineage hematopoiesis with maturation and 5% atypical plasma cells. No significant increase in lymphocytes was seen. The decision was made by the managing physician to monitor the patient. Three months later, laboratory studies showed an IgA of 3630 and a serum M-spike of 2.04 g/dL.

In February 2014, she presented to our clinic with constipation, nausea, weakness, diaphoresis and her serum creatinine was noted to be elevated at 1.3 mg/dL along with an IgA of 4590 mg/dL and a serum M-spike of 2.54 g/dL. She subsequently underwent a repeat bone marrow biopsy, which showed a variable cellular marrow with trilineage hematopoiesis and abnormal plasma cells constituting 21% of marrow cells. Fluorescent in situ hybridization performed on (FISH) CD38-selected plasma cells revealed a possibly unbalanced 13q rearrangement (extra 13q signal in 63/200 interphase nuclei). A repeat PET/CT scan showed widespread active adenopathy involving the bilateral neck, bilateral axillae and pre-pectoral regions, central abdominal adenopathy, retroperitoneal adenopathy, bilateral pelvic adenopathy (left greater than right), and inguinal adenopathy (left greater than right). Metabolic activity in these nodes had increased compared to the prior exam. A skeletal survey at that time revealed no definite lytic lesions.

She was subsequently started on CyBorD therapy (Cytoxan, 300 mg/m^2^ IV weekly, Bortezomib, 1.3 mg/m^2^ SQ weekly, and dexamethasone, 40 mg weekly). She completed two four-week cycles of this therapy with laboratory studies showing less than a partial response per IMWG criteria. A mixed response was noted on PET/CT. Therefore, the decision was made to pursue more aggressive induction therapy. She received two cycles of D-PACE (dexamethasone (40 mg/d days 1-4) with infusional cisplatin (10 mg/m^2^/d days 1-4), doxorubicin (10 mg/m^2^/d days 1-4), cyclophosphamide (400 mg/m^2^/d days 1-4), and etoposide (40 mg/m^2^/d days 1-4)) followed by one cycle of D-PACE with bortezomib (VD-PACE). At the conclusion of this therapy in August 2014, laboratory studies showed continued partial response, bone marrow biopsy showed less than 5% plasma cells, and PET/CT showed a mixed response.

In September 2014, she underwent stem cell collection and autologous stem cell transplant with a melphalan, 200 mg/m^2^, conditioning regimen. Her transplant admission was complicated by delayed count recovery requiring prolonged growth factor support. It was also complicated by persistent fevers with a presumptive diagnosis of colitis as well as persistent nausea/vomiting and several episodes of hemoptysis. Following discharge from the hospital, her course was complicated by persistent cytopenias requiring transfusion support and growth factor. A bone marrow biopsy on day 60 showed a variable cellular marrow with sparse polytypic plasma cells and a normal FISH panel. Laboratory studies from December 2014 showed several M-proteins in the gamma region with IgG lambda, monoclonal kappa, and IgA band without corresponding bound light chain. PET/CT scan showed a mixed response to therapy. 

**Figure 2:**
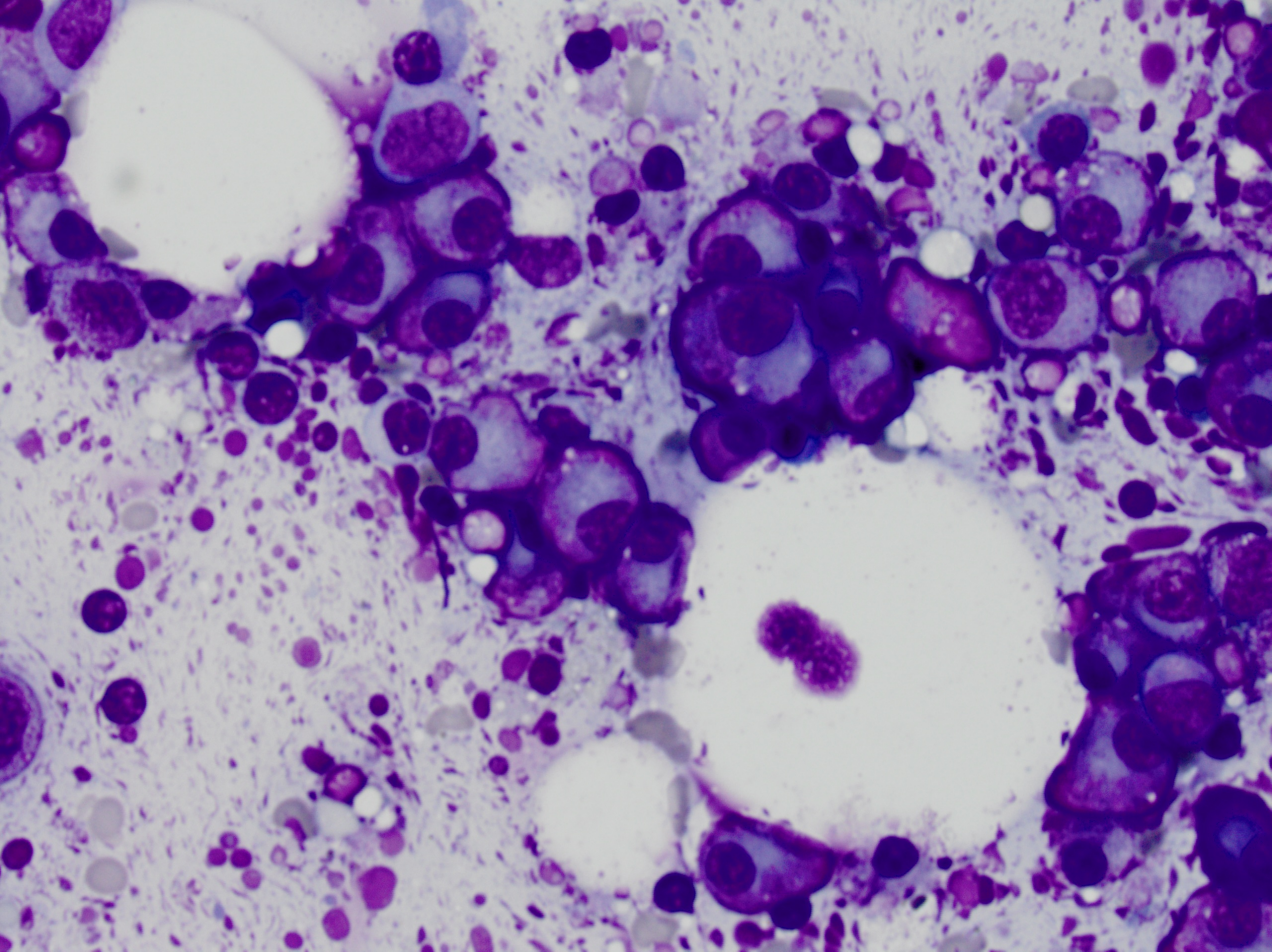
Bone marrow aspirate smear shows frequent clusters of abnormal plasma cells (600X magnification).

In January 2015, she had a recurrence of her original monoclonal protein with an IgA of 2469 mg/dL and serum M-spike of 2.56 g/dL. Bone marrow biopsy revealed myeloma involving 50-70% of the marrow with normal FISH testing (Figure 2). She remained pancytopenic, requiring transfusion support and growth factor support. At this time, a discussion was held with the patient regarding treatment options. She remained debilitated from the transplant, and as aggressive induction therapy followed by high-dose melphalan with autologous stem cell support had been unable to control her disease, she elected not to pursue additional chemotherapy. However, for palliative measures, the decision was made to initiate high-dose methylprednisolone as per Gertz, et al. [1]. The patient received methylprednisolone, 2 g IV 3x weekly from January 16 through March 11, 2015, when she was lost to follow-up and then re-presented April 6, 2015. At that time, she was transitioned to weekly dosing.

Over the next five months, she continued to receive weekly methylprednisolone. She became transfusion-independent, although she remained anemic (hemoglobin in the 10-11 g/dL range) and thrombocytopenic (platelets in the 30-40K range). Her performance status steadily improved, and she completed many items on her “bucket list”. Her myeloma labs were monitored on a monthly basis and demonstrated a steady decline in both the IgA level and serum M-spike. By September 2015, the IgA was 687 mg/dL and serum M-spike was 0.4 g/dL. A follow-up PET/CT showed a complete metabolic response to therapy. A bone marrow biopsy showed 1-5% plasma cells. Flow cytometry on the aspirate revealed a population of abnormal plasma cells (1%) expressing CD38, cytoplasmic kappa light chain, CD19, and was negative for CD138, CD56, CD117, and CD28. Given this remarkable response to therapy, coupled with the patient’s improved performance status and increasingly cushingoid appearance from the high-dose steroids, the decision was made to discontinue methylprednisolone and start lenalidomide with weekly dexamethasone.

## Discussion

The malignant plasma cells in multiple myeloma are highly dependent on the bone marrow microenvironment. However, extramedullary disease in the form of soft tissue plasmacytomas can occur either through direct extension from bony lesions or by hematogenous spread. The incidence of extramedullary disease at the time of presentation has been reported to be anywhere from 2-20% [2-3]. Extramedullary disease has been associated with high-risk myeloma and with shorter progression-free and overall survival rates [2]. Whether patients who present with only nodal involvement as the site of extramedullary disease have a different prognosis than other extramedullary sites is not known.

The entity of primary lymph node plasmacytomas (PLNPs) has been described as having a male predominance, associated with IgG kappa paraproteinemia, and no progression to myeloma [4]. In addition, the histological features of PLNPs were reported to be more mature than reactive plasmacytosis but less immature than in myeloma [4]. Shao, et al. described nine cases of IgA plasmacytomas presenting in the lymph nodes [5]. Two-thirds of patients were found to have associated immune dysfunction, including HIV, T-cell deficiency, autoantibodies, arthritis, or Sjogren syndrome. These patients appeared to have an indolent disease and none progressed to myeloma.

In the case reported here, the patient not only had widespread lymph node involvement but also bone marrow involvement; thus, the diagnosis was consistent with myeloma with extramedullary nodal disease. Interestingly, the bone marrow involvement was minimal at the time of diagnosis but was significantly involved at the time of disease progression post-transplantation. One could speculate that the “re-setting” of her bone marrow post-transplant may have provided her disease with a more favorable microenvironment. The fact that her disease displayed minimal responsiveness to very aggressive chemotherapy but was then responsive to a prolonged course of high-dose steroids is quite unusual.

It has long been recognized that multiple myeloma is sensitive to corticosteroids. A response rate of 43% was observed with dexamethasone as a single agent for newly diagnosed myeloma [6]. However, despite the fact that corticosteroids have been used to treat myeloma for 50 years, the mechanisms underlying sensitivity or resistance remain incompletely understood. Although there are now many classes of drugs available for the treatment of myeloma, including proteasome inhibitors, immunomodulatory drugs, histone deacetylase inhibitors, and more traditional agents, such as alkylators and anthracyclines, primary refractoriness or development of resistance remains a significant problem.

## Conclusions

This case demonstrates that high-dose steroids may provide not only palliative benefit but also disease control, even in the setting of significant extramedullary involvement and refractoriness to more standard anti-myeloma therapies. In the future, the utilization of methodologies, such as expression arrays and next-generation sequencing, to analyze the genetic composition of extramedullary disease in comparison with marrow disease may provide insight into not only the pathophysiology of the disease but also into mechanisms underlying drug resistance.
